# 4-Cyano­pyridinium hydrogen sulfate

**DOI:** 10.1107/S1600536812027304

**Published:** 2012-06-23

**Authors:** Ying-Chun Wang

**Affiliations:** a College of Chemistry and Chemical Engineering, Southeast University, Nanjing 210096, People’s Republic of China

## Abstract

All non-H atoms of the cation of the title salt, C_6_H_5_N_2_^+^·HSO_4_^−^, are essentially coplanar [r.m.s. deviation = 0.005 (1) Å] . In the crystal, N—H⋯O and O—H⋯O hydrogen bonds and weak C—H⋯O and C—H⋯N inter­actions link the mol­ecules into a two-dimensional network parallel to the (001) plane. Weak π–π stacking inter­actions between the pyridine rings of neighbouring mol­ecules further stabilize the structure [centroid–centroid distance = 3.785 (1) Å].

## Related literature

For materials which display ferroelectric–paraelectric phase transitions, see: Chen *et al.* (2001 ▶); Huang *et al.* (1999 ▶); Zhang *et al.* (2001 ▶); For the structures and properties of related compounds, see: Wang *et al.* (2002 ▶); Xue *et al.* (2002 ▶); Ye *et al.* (2008 ▶).
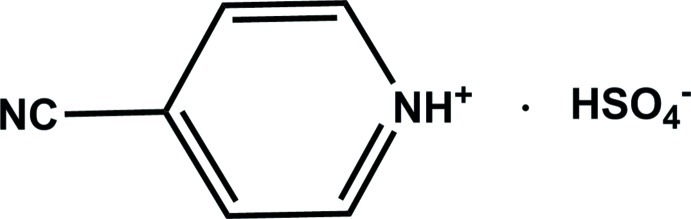


## Experimental

### 

#### Crystal data

C_6_H_5_N_2_^+^·HSO_4_^−^
*M* *_r_* = 202.19
Orthorhombic, 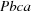

*a* = 14.2959 (12) Å
*b* = 7.8817 (8) Å
*c* = 14.4280 (13) Å
*V* = 1625.7 (3) Å^3^
*Z* = 8
Mo *K*α radiation
μ = 0.38 mm^−1^
*T* = 123 K
0.10 × 0.05 × 0.05 mm

#### Data collection

Rigaku Mercury2 diffractometer
Absorption correction: multi-scan (*CrystalClear*; Rigaku, 2005 ▶) *T*_min_ = 0.910, *T*_max_ = 1.000
16314 measured reflections
1854 independent reflections
1795 reflections with *I* > 2σ(*I*)
*R*_int_ = 0.032

#### Refinement

*R*[*F* ^2^ > 2σ(*F* ^2^)] = 0.034
*wR*(*F* ^2^) = 0.092
*S* = 1.19
1854 reflections
118 parameters
2 restraints
H-atom parameters constrained
Δρ_max_ = 0.33 e Å^−3^
Δρ_min_ = −0.40 e Å^−3^

### 

Data collection: *CrystalClear* (Rigaku, 2005 ▶); cell refinement: *CrystalClear*; data reduction: *CrystalClear*; program(s) used to solve structure: *SHELXS97* (Sheldrick, 2008 ▶); program(s) used to refine structure: *SHELXL97* (Sheldrick, 2008 ▶); molecular graphics: *SHELXTL* (Sheldrick, 2008 ▶); software used to prepare material for publication: *SHELXTL*.

## Supplementary Material

Crystal structure: contains datablock(s) I, global. DOI: 10.1107/S1600536812027304/jj2139sup1.cif

Structure factors: contains datablock(s) I. DOI: 10.1107/S1600536812027304/jj2139Isup2.hkl

Supplementary material file. DOI: 10.1107/S1600536812027304/jj2139Isup3.cml

Additional supplementary materials:  crystallographic information; 3D view; checkCIF report

## Figures and Tables

**Table 1:** Hydrogen-bond geometry (Å, °)

*D*—H⋯*A*	*D*—H	H⋯*A*	*D*⋯*A*	*D*—H⋯*A*
N1—H1⋯O4^i^	0.89	1.87	2.7123 (16)	157
O2—H2⋯O1^ii^	0.82	1.79	2.5859 (15)	165
C1—H1*A*⋯O1^i^	0.93	2.50	3.2681 (19)	139
C2—H2*A*⋯O2^iii^	0.93	2.43	3.3279 (19)	162
C4—H4*A*⋯O3	0.93	2.43	3.2189 (19)	143
C5—H5*A*⋯O3^iv^	0.93	2.57	3.3192 (19)	138
C5—H5*A*⋯N2^v^	0.93	2.53	3.211 (2)	130

Symmetry codes: (i) 
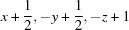
; (ii) 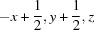; (iii) 
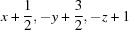
; (iv) 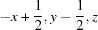; (v) 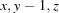.

**Fig. 1.**
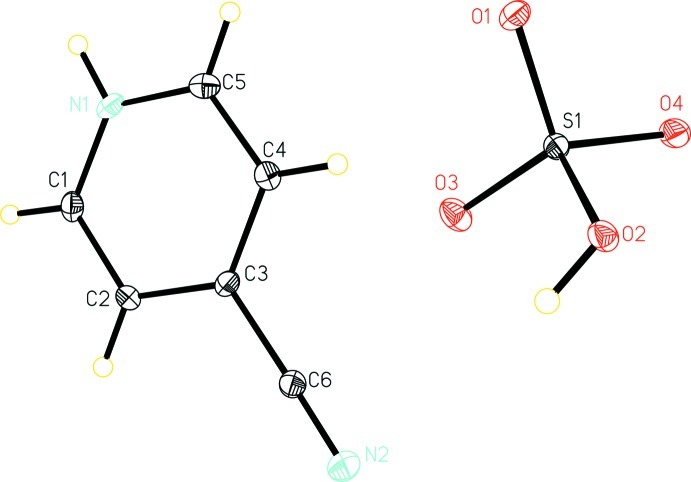
Molecular structure of the title compound showing the atom labeling scheme and 50% probability displacement ellipsoids for one cation-anion unit and bimolecular unit in the asymmetric unit.

**Fig. 2.**
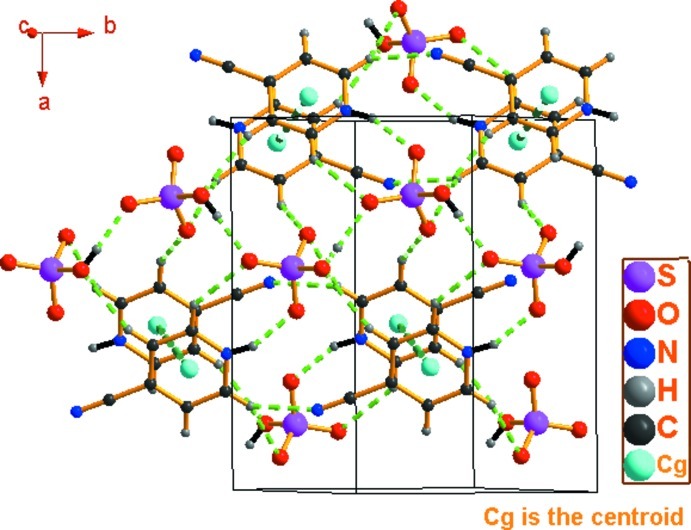
Crystal packing of the title compound viewed along the c axis showing N—H···O and O—H···O hydrogen bonds (dotted lines), weak C—H···O, C—H···N intermolecular interactions (dotted lines) and weak π—π stacking interactions (dashed lines).

## supplementary crystallographic information

### Comment

Simple organic salts containing strong intrermolecular H-bonds have attracted attention as materials which display ferroelectric-paraelectric phase transitions (Chen *et al.*, 2001; Huang, *et al.* 1999; Zhang, *et al.* 2001). In an effort to obtain phase transition crystals of organic salts, various organic molecules have been studied with a series of new crystal materials (Wang *et al.*, 2002; Xue, *et al.* 2002; Ye *et al.*, 2008). Herewith, we present the synthesis and crystal structure of the title compound, C_6_H_5_N_2_^+^.HSO_4_^-^,(I).

The asymmetric unit of (I) is comprised of one 4-cyanopyridinium cation and one HSO_4_^-^ anion (Fig. 1). All non-H atoms of the cation are essentially coplanar [r.m.s. deviation = 0.005 (1)Å ]. Bond lengths and angles in each of these units are in normal ranges.

In the crystal, N—H···O hydrogen bonds and weak C—H···O and C—H···N intermolecular interactions bring the organic molecules into a 2D network (Table 1, Fig. 2). In addition, weak π–π stacking interactions between the pyridine rings of neighbouring organic molecules further stabilize the structure (Cg···Cg = 3.785 (1)Å, with Cg being the centriod of the pyridine ring).

### Experimental

Isonicotinonitrile (10 mmol) and H_2_SO_4_ (1.0 mL, 10mmol/L) and ethanol (50 mL) were added into a 100mL flask. The mixture was stirred at 60°C for 2 h. The precipitate was then filtrated. Colourless crystals suitable for X-ray diffraction were obtained by slow evaporation of the solution.

### Refinement

H1, and H2 were refined freely. In the last stages of the refinement these atoms were restrained with N1—H1 = 0.89 (2)Å and O2—H2 = 0.82 (2)Å with *U_iso_*(H) = 1.2*U_eq_*(N) and *U_iso_*(H)=1.5*U_eq_*(O). All the remaining H atoms attached to C atoms were placed in calculated positions and then refined using the riding model with C—H lengths of 0.93 Å (CH). The isotropic displcement parameers for these atoms were set to 1.2 (CH) times *U*_eq_ of the parent atom.

### Crystal data

**Table:**

C_6_H_5_N_2_^+^·HSO_4_^−^	*F*(000) = 832
*M**_r_* = 202.19	*D*_x_ = 1.652 Mg m^−^^3^
Orthorhombic, *P**b**c**a*	Mo *K*α radiation, λ = 0.71073 Å
Hall symbol: -P 2ac 2ab	Cell parameters from 1854 reflections
*a* = 14.2959 (12) Å	θ = 2.9–27.5°
*b* = 7.8817 (8) Å	µ = 0.38 mm^−^^1^
*c* = 14.4280 (13) Å	*T* = 123 K
*V* = 1625.7 (3) Å^3^	Block, colorless
*Z* = 8	0.10 × 0.05 × 0.05 mm

### Data collection

**Table:**

Rigaku Mercury2 diffractometer	1854 independent reflections
Radiation source: fine-focus sealed tube	1795 reflections with *I* > 2σ(*I*)
Graphite monochromator	*R*_int_ = 0.032
Detector resolution: 13.6612 pixels mm^-1^	θ_max_ = 27.5°, θ_min_ = 2.9°
CCD profile fitting scans	*h* = −18→18
Absorption correction: multi-scan (*CrystalClear*; Rigaku, 2005)	*k* = −10→10
*T*_min_ = 0.910, *T*_max_ = 1.000	*l* = −18→18
16314 measured reflections	

### Refinement

**Table:**

Refinement on *F*^2^	Primary atom site location: structure-invariant direct methods
Least-squares matrix: full	Secondary atom site location: difference Fourier map
*R*[*F*^2^ > 2σ(*F*^2^)] = 0.034	Hydrogen site location: inferred from neighbouring sites
*wR*(*F*^2^) = 0.092	H-atom parameters constrained
*S* = 1.19	*w* = 1/[σ^2^(*F*_o_^2^) + (0.045*P*)^2^ + 0.7898*P*] where *P* = (*F*_o_^2^ + 2*F*_c_^2^)/3
1854 reflections	(Δ/σ)_max_ < 0.001
118 parameters	Δρ_max_ = 0.33 e Å^−^^3^
2 restraints	Δρ_min_ = −0.40 e Å^−^^3^

### Special details

**Table:**

Geometry. All esds (except the esd in the dihedral angle between two l.s. planes) are estimated using the full covariance matrix. The cell esds are taken into account individually in the estimation of esds in distances, angles and torsion angles; correlations between esds in cell parameters are only used when they are defined by crystal symmetry. An approximate (isotropic) treatment of cell esds is used for estimating esds involving l.s. planes.
Refinement. Refinement of F^2^ against ALL reflections. The weighted R-factor wR and goodness of fit S are based on F^2^, conventional R-factors R are based on F, with F set to zero for negative F^2^. The threshold expression of F^2^ > 2sigma(F^2^) is used only for calculating R-factors(gt) etc. and is not relevant to the choice of reflections for refinement. R-factors based on F^2^ are statistically about twice as large as those based on F, and R- factors based on ALL data will be even larger.

### Fractional atomic coordinates and isotropic or equivalent isotropic displacement parameters (Å^2^)

**Table:**

	*x*	*y*	*z*	*U*_iso_*/*U*_eq_	
S1	0.17080 (2)	0.57874 (4)	0.66263 (2)	0.01293 (13)	
O1	0.18628 (8)	0.43049 (13)	0.72165 (8)	0.0186 (2)	
N1	0.48319 (9)	0.22311 (16)	0.39112 (9)	0.0182 (3)	
H1	0.4995	0.1142	0.3932	0.022*	
C1	0.53425 (11)	0.3477 (2)	0.35266 (11)	0.0198 (3)	
H1A	0.5907	0.3226	0.3236	0.024*	
O2	0.16838 (7)	0.73568 (14)	0.72925 (7)	0.0165 (2)	
H2	0.2140	0.7945	0.7167	0.025*	
N2	0.35791 (11)	0.85408 (19)	0.40783 (12)	0.0317 (4)	
C2	0.50280 (11)	0.5123 (2)	0.35638 (11)	0.0182 (3)	
H2A	0.5370	0.6000	0.3297	0.022*	
O3	0.24390 (8)	0.60728 (15)	0.59562 (7)	0.0200 (3)	
C3	0.41822 (10)	0.54447 (18)	0.40125 (10)	0.0154 (3)	
O4	0.07646 (8)	0.57536 (13)	0.62351 (8)	0.0190 (3)	
C4	0.36712 (11)	0.41442 (19)	0.44145 (10)	0.0173 (3)	
H4A	0.3113	0.4365	0.4724	0.021*	
C5	0.40147 (11)	0.2508 (2)	0.43428 (10)	0.0182 (3)	
H5A	0.3681	0.1605	0.4593	0.022*	
C6	0.38364 (11)	0.7175 (2)	0.40508 (12)	0.0204 (3)	

### Atomic displacement parameters (Å^2^)

**Table:**

	*U*^11^	*U*^22^	*U*^33^	*U*^12^	*U*^13^	*U*^23^
S1	0.0118 (2)	0.0120 (2)	0.0150 (2)	−0.00042 (11)	0.00102 (12)	−0.00076 (12)
O1	0.0191 (5)	0.0144 (5)	0.0223 (6)	0.0046 (4)	0.0025 (4)	0.0028 (4)
N1	0.0220 (6)	0.0129 (6)	0.0196 (6)	0.0040 (5)	−0.0051 (5)	−0.0009 (5)
C1	0.0139 (7)	0.0225 (8)	0.0230 (7)	0.0022 (6)	−0.0007 (5)	−0.0026 (6)
O2	0.0151 (5)	0.0146 (5)	0.0198 (6)	−0.0026 (4)	0.0034 (4)	−0.0042 (4)
N2	0.0259 (7)	0.0183 (8)	0.0511 (10)	0.0021 (6)	0.0009 (7)	−0.0049 (7)
C2	0.0149 (7)	0.0166 (7)	0.0231 (7)	−0.0024 (5)	−0.0004 (6)	0.0013 (6)
O3	0.0189 (5)	0.0220 (6)	0.0192 (5)	−0.0039 (4)	0.0059 (4)	−0.0035 (4)
C3	0.0164 (7)	0.0134 (7)	0.0164 (7)	0.0019 (5)	−0.0045 (5)	−0.0025 (5)
O4	0.0157 (5)	0.0164 (5)	0.0249 (6)	−0.0028 (4)	−0.0049 (4)	0.0013 (4)
C4	0.0164 (7)	0.0189 (8)	0.0166 (7)	−0.0007 (5)	0.0005 (5)	−0.0028 (5)
C5	0.0242 (8)	0.0163 (7)	0.0142 (7)	−0.0026 (6)	−0.0017 (6)	0.0003 (6)
C6	0.0168 (7)	0.0163 (8)	0.0281 (8)	−0.0005 (6)	−0.0022 (6)	−0.0034 (6)

### Geometric parameters (Å, º)

**Table:**

S1—O3	1.4414 (11)	O2—H2	0.8198
S1—O4	1.4622 (11)	N2—C6	1.138 (2)
S1—O1	1.4626 (11)	C2—C3	1.395 (2)
S1—O2	1.5669 (11)	C2—H2A	0.9300
N1—C5	1.342 (2)	C3—C4	1.386 (2)
N1—C1	1.343 (2)	C3—C6	1.452 (2)
N1—H1	0.8902	C4—C5	1.384 (2)
C1—C2	1.375 (2)	C4—H4A	0.9300
C1—H1A	0.9300	C5—H5A	0.9300
			
O3—S1—O4	114.37 (7)	C1—C2—C3	118.23 (14)
O3—S1—O1	113.92 (7)	C1—C2—H2A	120.9
O4—S1—O1	110.48 (6)	C3—C2—H2A	120.9
O3—S1—O2	107.71 (6)	C4—C3—C2	121.13 (14)
O4—S1—O2	103.34 (6)	C4—C3—C6	119.96 (14)
O1—S1—O2	106.07 (6)	C2—C3—C6	118.91 (14)
C5—N1—C1	123.08 (13)	C5—C4—C3	118.09 (14)
C5—N1—H1	111.7	C5—C4—H4A	121.0
C1—N1—H1	125.2	C3—C4—H4A	121.0
N1—C1—C2	119.75 (14)	N1—C5—C4	119.71 (14)
N1—C1—H1A	120.1	N1—C5—H5A	120.1
C2—C1—H1A	120.1	C4—C5—H5A	120.1
S1—O2—H2	107.1	N2—C6—C3	178.92 (18)

### Hydrogen-bond geometry (Å, º)

**Table:**

*D*—H···*A*	*D*—H	H···*A*	*D*···*A*	*D*—H···*A*
N1—H1···O4^i^	0.89	1.87	2.7123 (16)	157
O2—H2···O1^ii^	0.82	1.79	2.5859 (15)	165
C1—H1*A*···O1^i^	0.93	2.50	3.2681 (19)	139
C2—H2*A*···O2^iii^	0.93	2.43	3.3279 (19)	162
C4—H4*A*···O3	0.93	2.43	3.2189 (19)	143
C5—H5*A*···O3^iv^	0.93	2.57	3.3192 (19)	138
C5—H5*A*···N2^v^	0.93	2.53	3.211 (2)	130

Symmetry codes: (i) *x*+1/2, −*y*+1/2, −*z*+1; (ii) −*x*+1/2, *y*+1/2, *z*; (iii) *x*+1/2, −*y*+3/2, −*z*+1; (iv) −*x*+1/2, *y*−1/2, *z*; (v) *x*, *y*−1, *z*.
